# Antimicrobial use in Canadian acute-care hospitals: Findings from three national point-prevalence surveys between 2002 and 2017

**DOI:** 10.1017/ice.2021.519

**Published:** 2022-11

**Authors:** Jennifer J. Liang, Wallis Rudnick, Robyn Mitchell, James Brooks, Kathryn Bush, John Conly, Jennifer Ellison, Charles Frenette, Lynn Johnston, Christian Lavallée, Allison McGeer, Dominik Mertz, Linda Pelude, Michelle Science, Andrew Simor, Stephanie Smith, Paula Stagg, Kathryn N. Suh, Nisha Thampi, Daniel J.G. Thirion, Joseph Vayalumkal, Alice Wong, Geoffrey Taylor, for the Canadian Nosocomial Infection Surveillance Program

**Affiliations:** 1 Public Health Agency of Canada, Ottawa, Ontario, Canada; 2 Alberta Health Services, Calgary, Alberta, Canada; 3 University of Calgary, Calgary, Alberta, Canada; 4 McGill University Health Centre, Montréal, Québec, Canada; 5 Queen Elizabeth II Health Sciences Centre, Halifax, Nova Scotia, Canada; 6 Hôpital Maisonneuve-Rosemont, Montréal, Québec, Canada; 7 Sinai Health Systems, Toronto, Ontario, Canada; 8 Department of Medicine, McMaster University, Hamilton, Ontario, Canada; 9 Hamilton Health Sciences, Hamilton, Ontario, Canada; 10 Hospital for Sick Children, Toronto, Ontario, Canada; 11 Sunnybrook Health Sciences Centre, Toronto, Ontario, Canada; 12 University of Alberta Hospital, Edmonton, Alberta, Canada; 13 Western Memorial Regional Hospital, Corner Brook, Newfoundland, Canada; 14 The Ottawa Hospital, Ottawa, Ontario, Canada; 15 Children’s Hospital of Eastern Ontario, Ottawa, Ontario, Canada; 16 Université de Montréal, Montréal, Québec, Canada; 17 Alberta Children’s Hospital, Calgary, Alberta, Canada; 18 Royal University Hospital, Saskatoon, Saskatchewan, Canada

**Keywords:** antimicrobial use, point prevalence surveys, hospital epidemiology, healthcare-associated infections

## Abstract

**Objectives:**

The Canadian Nosocomial Infection Surveillance Program conducted point-prevalence surveys in acute-care hospitals in 2002, 2009, and 2017 to identify trends in antimicrobial use.

**Methods:**

Eligible inpatients were identified from a 24-hour period in February of each survey year. Patients were eligible (1) if they were admitted for ≥48 hours or (2) if they had been admitted to the hospital within a month. Chart reviews were conducted. We calculated the prevalence of antimicrobial use as follows: patients receiving ≥1 antimicrobial during survey period per number of patients surveyed × 100%.

**Results:**

In each survey, 28−47 hospitals participated. In 2002, 2,460 (36.5%; 95% CI, 35.3%−37.6%) of 6,747 surveyed patients received ≥1 antimicrobial. In 2009, 3,566 (40.1%, 95% CI, 39.0%−41.1%) of 8,902 patients received ≥1 antimicrobial. In 2017, 3,936 (39.6%, 95% CI, 38.7%−40.6%) of 9,929 patients received ≥1 antimicrobial. Among patients who received ≥1 antimicrobial, penicillin use increased 36.8% between 2002 and 2017, and third-generation cephalosporin use increased from 13.9% to 18.1% (*P* < .0001). Between 2002 and 2017, fluoroquinolone use decreased from 25.7% to 16.3% (*P* < .0001) and clindamycin use decreased from 25.7% to 16.3% (*P* < .0001) among patients who received ≥1 antimicrobial. Aminoglycoside use decreased from 8.8% to 2.4% (*P* < .0001) and metronidazole use decreased from 18.1% to 9.4% (*P* < .0001). Carbapenem use increased from 3.9% in 2002 to 6.1% in 2009 (*P* < .0001) and increased by 4.8% between 2009 and 2017 (*P* = .60).

**Conclusions:**

The prevalence of antimicrobial use increased between 2002 and 2009 and then stabilized between 2009 and 2017. These data provide important information for antimicrobial stewardship programs.

Antimicrobial resistance is a major global health problem threatening the ability to treat and prevent infections. Understanding the trends and magnitude of antimicrobial use in hospitals is important because overuse of antimicrobials is a major contributor to antimicrobial resistance and hospitals are a major source of antimicrobial resistant pathogens.^1^

Data on antimicrobial use within Canadian hospitals are limited. A subset of Canadian hospitals within the Canadian Nosocomial Infection Surveillance Program (CNISP) network reported a 12% reduction in dispensed annual hospital-level antimicrobials among adult inpatients from 2009 to 2016; however, no patient-level data were collected.^2^

Point-prevalence surveys can provide patient-level antimicrobial use; these data enable comparisons between patient characteristics and hospital locations. By understanding the burden and trends of antimicrobial use among different patient populations, antimicrobial stewardship programs can target and develop interventions for specific patient populations and antimicrobial agents. By providing national benchmarks and trends over time, point-prevalence surveys enable comparisons between hospitals.

The CNISP network conducted point-prevalence surveys in acute-care hospitals in 2002, 2009, and 2017 to identify trends in healthcare-associated infections (HAIs) and antimicrobial use among inpatients. As described elsewhere,^3^ among all HAIs, the percentage of *S. aureus* isolates that were methicillin resistant remained consistent across the surveys (28%–31%), whereas the percentage of *Enterococcus* isolates that were vancomycin resistant increased from 1.9% (2002) to 8.2% (2017). Infections due to carbapenemase-producing organisms were rare (0.1% of HAIs). We analyzed the survey data to describe trends in antimicrobial use prevalence by age group and ward type.

## Methods

### Study design

One-day point-prevalence surveys were conducted at Canadian acute-care hospitals in February 2002, 2009, and 2017. Survey methodology has been published elsewhere.^3^

### Data sources and study population

Hospitals participated as members of the CNISP network. The CNISP is a collaboration of the Public Health Agency of Canada and the Canadian Hospital Epidemiology Committee, a subcommittee of the Association of Medical Microbiology and Infectious Disease Canada.^4^

On the days when the surveys were conducted, patients were eligible for inclusion (1) if they were admitted to the participating hospital for ≥48 hours in total or (2) if they had been previously admitted to the survey hospital within the last month (for any length of time). We excluded patients admitted to long-term care, maternity, mental health, day surgery, or rehabilitation units.

### Data collection

Eligible patients were identified using hospital census data on a specified day and time in February of each survey year to minimize seasonal variation. The survey period included the 24 hours after the census was taken. Trained infection prevention and control professionals collected patient information by reviewing patient charts and completing a standardized questionnaire. Collected information included demographic data, admission date, ward where the patient was located during the survey period, and information on HAIs. Antimicrobials received (≥1 dose) during the survey period were identified by chart review and were classified by antimicrobial drug class.

The list of parenteral and oral antimicrobials that were included in the survey and their classifications are provided in Supplementary Table 1 (online). Clindamycin, third-generation cephalosporins, fluoroquinolones, and penicillin combinations with β-lactamase inhibitors were classified as ‘high-risk’ antimicrobials for *Clostridiodes difficile* infection (CDI). Information on administration route was not collected. Questionnaires were submitted to the CNISP for data entry and verification.

### Data analysis

We analyzed the data using SAS version 9.4 software (SAS Institute, Cary, NC). We calculated the prevalence of antimicrobial use as the percentage of patients receiving ≥1 antimicrobial during the survey period over the total number of patients surveyed. We used Poisson regression with survey year as a categorical exposure variable to calculate the differences in AMU prevalence. We used generalized estimating equations with robust standard errors to account for clustering by hospital and to calculate *P* values. We considered a 2-sided *P* value ≤.05 to be statistically significant. Because participating hospitals changed between surveys and this could have affected trends, we conducted a sensitivity analysis using only the 18 hospitals that participated in all 3 surveys.

## Results

### Description of participating hospitals

Each survey included 28–47 hospitals and 6,747–9,929 surveyed patients (Table 1). Across the surveys, participating hospitals remained similar with respect to geography, bed size, hospital type and specialized services as previously described.^3^ In 2002 and 2009, hospitals from 9 provinces participated in the point-prevalence surveys, with an additional province participating in 2017. The percentage of patients in medical-surgical wards decreased from 72% in 2002 to 57% in 2017, and the percentage of patients in intensive care units increased slightly from 11% in 2002 to 13% in 2017.

**Table 1.** Selected Characteristics of Hospitals Participating in the Point-Prevalence Surveys (2002, 2009 and 2017)

Selected Survey Characteristics^a^		2002	2009	2017
No. of hospitals surveyed		28	39	47
No. of patients surveyed		6,747	8,902	9,929
No. of surveyed patients who received ≥1 antimicrobial		2,460	3,566	3,936
**Patient location**	% patients in medical-surgical wards	72.4	66.7	57.1
	% patients in ICUs^b^	10.6	11.5	12.7
	% in other locations^c^	17.0	21.8	30.2

Note. ICU, intensive care unit.

a For a detailed description of survey characteristics across the 3 surveys, see Mitchell et al.^3^

b Includes adult ICU, neonatal ICU, and pediatric ICU.

c Other locations includes critical/coronary care (not ICU), gynecology–obstetrics, hematology–oncology–bone marrow transplant, solid organ transplant, pediatrics, trauma/burn unit and others not listed.

### Overall antimicrobial use

**Fig. 1.**
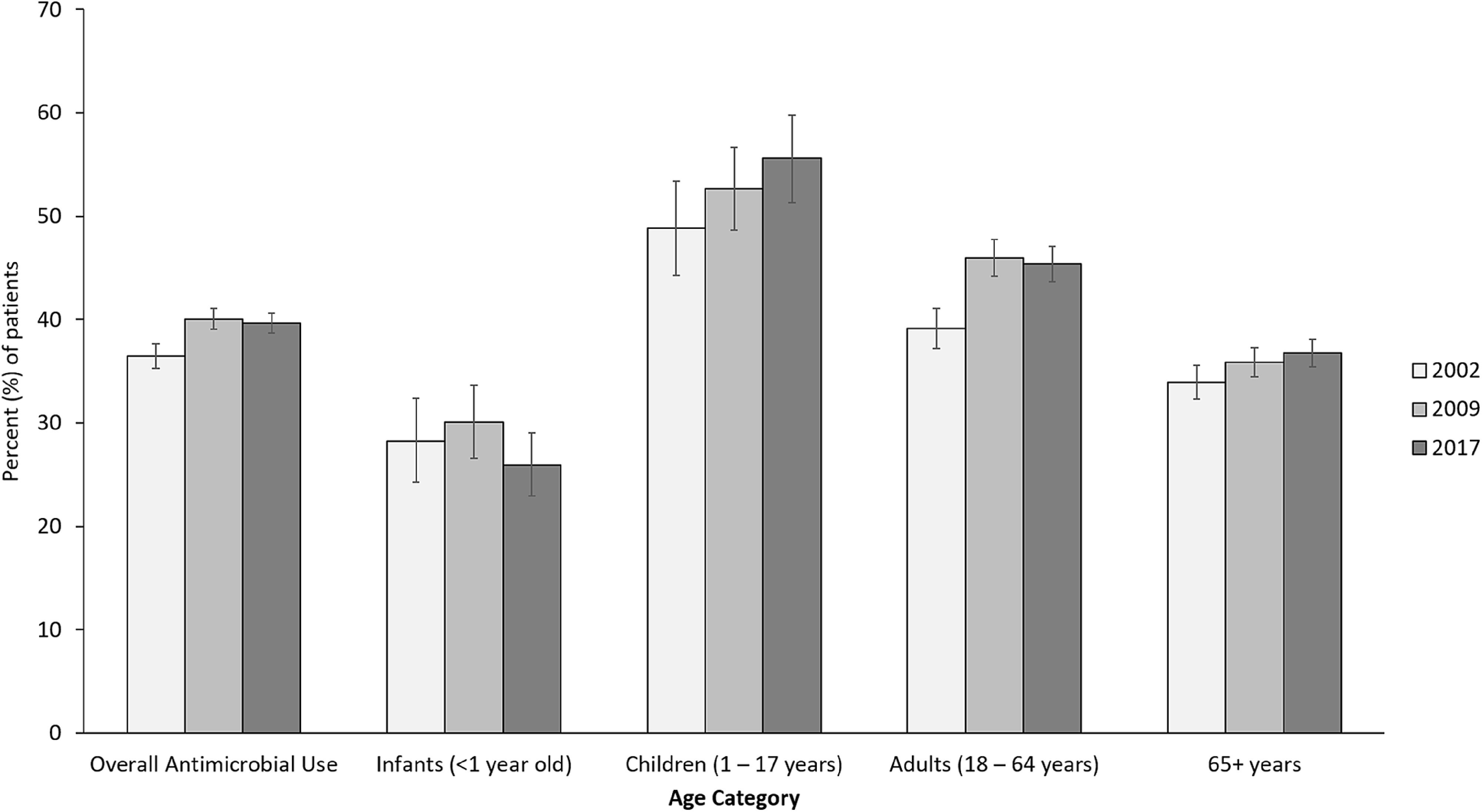
Prevalence of antimicrobial use in 2002, 2009, and 2017 (overall and by age category with 95% confidence intervals).

The percentages of surveyed patients who received ≥1 antimicrobial were 36.5% (95% CI, 35.3%−37.6%) in 2002, 40.1% (95% CI, 39.0%−41.1%) in 2009, and 39.6% (95% CI, 38.7%−40.6%) in 2017 (Fig. 1). Overall, the prevalence of antimicrobial use significantly increased by 9.9% (*P* = .0007) from 2002 to 2009 but remained stable (1.1% decrease; *P* = .80) from 2009 to 2017. We observed an increase in antimicrobial use among children (aged 1−17 years) and patients aged >65 years, although neither change was significant (Fig. 1). Among adults aged 18−64 years, antimicrobial use increased by 17.5% (*P* = .002) from 2002 to 2009 and decreased 1.3% (*P* = .80) from 2009 to 2017. In infants, antimicrobial use increased by 6.6% (*P* = .60) from 2002 to 2009 and decreased by 13.7% (*P* = .07) between 2009 and 2017.

A small set of antimicrobials (daptomycin, tigecycline, micafungin, posaconazole, anidulafungin, ceftolozane/tazobactam, and ceftazidime/avibactam) were not approved for use in 2002 but were captured on subsequent surveys. In aggregate these antimicrobials represented 0.2% and 1.0% of use in 2009 and 2017, respectively.

### Antimicrobial use by hospital ward

The prevalence of antimicrobial use among patients on mixed medical-surgical wards increased from 33.4% (95% CI, 32.1%−34.8%) in 2002 to 36.6% (95% CI, 35.5%−37.8%) in 2009 and to 37.7% (95% CI, 36.5%−38.9%) in 2017. From 2002 to 2017, there was a 12.8% (*P* = .01) relative increase in antimicrobial use among patients admitted to mixed medical-surgical wards.

Among adult patients on ICU wards, the percentages of patients who received ≥1 antimicrobial were 67% in 2002 and 2009 and 60% in 2017. Among adult patients on ICU wards who received ≥1 antimicrobial, the percentage of patients receiving only 1 antimicrobial was 38.8% (95% CI, 31.0%−48.6%) in 2002, increasing to 54.4% (95% CI, 47.0%−63.0%) in 2009 and to 58.8% (95% CI, 51.3%−67.4%) in 2017. This increase was driven by increases in the prevalence of patients receiving piperacillin-tazobactam, ceftriaxone, meropenem, and trimethoprim-sulfamethoxazole as monotherapy.

Patients in adult ICUs were more likely to have received an antimicrobial compared to adult non-ICU patients (60% vs 39% in 2017; *P* < .0001). Vancomycin (oral and intravenous combined), carbapenems, and antifungal agents were received more frequently by adult ICU patients (18%, 17%, and 13%, respectively) compared to non-ICU adult inpatients (12%, 6%, and 7%, respectively) (Fig. 4). The prevalence of fluoroquinolone use was higher on adult non-ICU wards (18.8%; 95% CI, 17.3%−20.4%) than on adult ICU wards (11.9%; 95%CI 8.8%−16.2%).

### Antimicrobial use by class and category

**Fig. 2.**
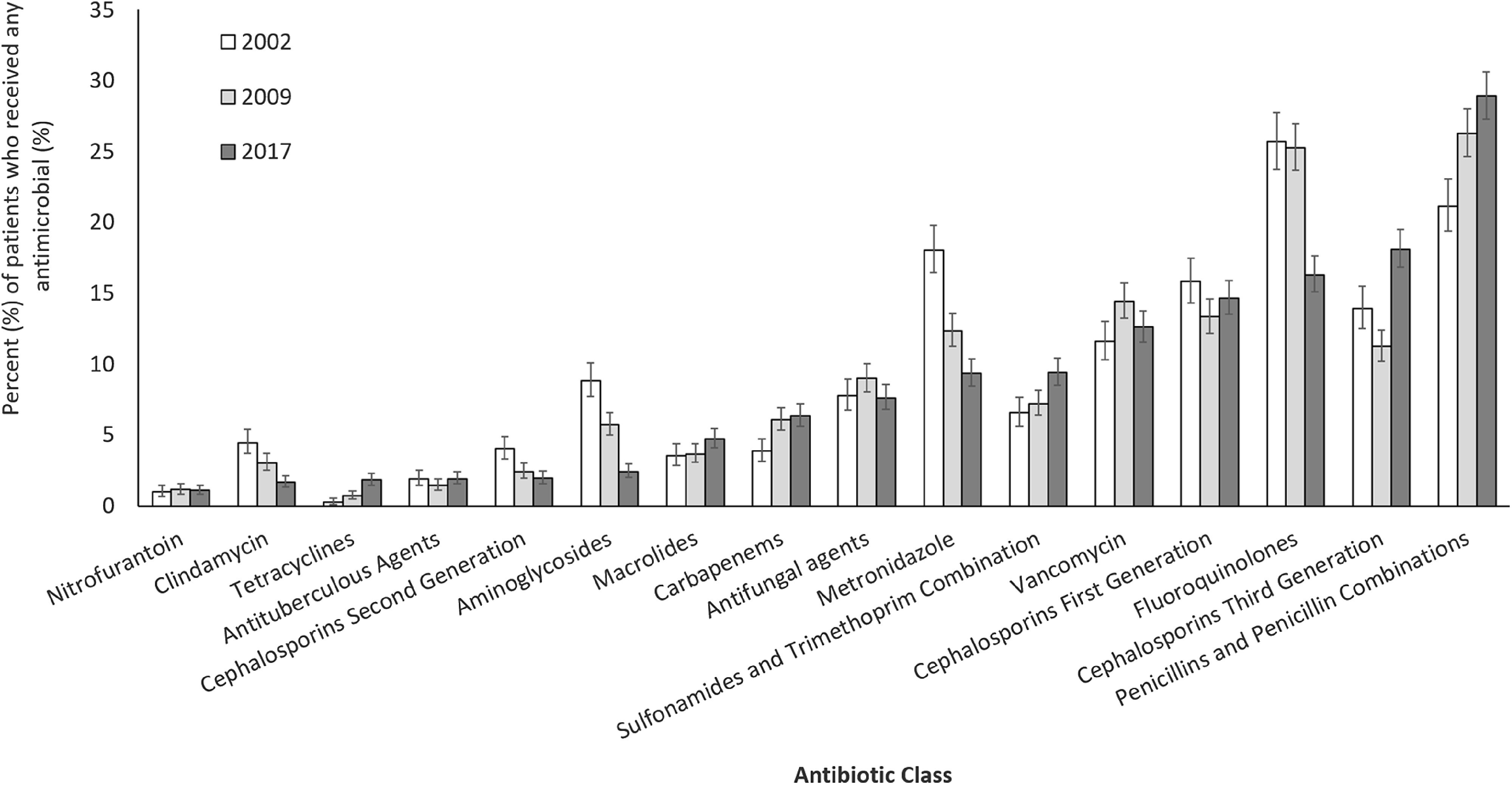
Percentage of patients who received selected antibiotic classes out of all patients who received any antimicrobial agent in 2002, 2009, and 2017 with 95% confidence intervals; antimicrobial classes ordered left to right from least to most frequently used in 2017.

Among patients who received ≥1 antimicrobial, the classes of antimicrobials used changed over time (Fig. 2). We detected a 36.8% increase in the use of penicillin and penicillin combinations among patients who received ≥1 antimicrobial between 2002 and 2017. Of penicillin and penicillin combination-class agents, the proportion accounted for by piperacillin-tazobactam increased from 16.9% (95% CI, 13.7%−20.9%) in 2002 to 44.2% (95% CI, 40.1%−48.7%) in 2009 to 56.0% (95% CI, 51.8%−60.5%) in 2017 (*P* < .0001) (see Supplementary Fig. 1 online for trends among specific penicillin antibiotics). Among patients who received ≥1 antimicrobial, the prevalence of patients who received ≥1 antimicrobial with a high risk of CDI was 47.8% in 2002, 51.1% in 2009, and 55.8% in 2017. The prevalence of patients who received both antipseudomonal and anti-MRSA antimicrobials was 4.4% (108 of 2,460) in 2002, 6.1% (216 of 3,566) in 2009, and 5.0% (196 of 3,936) in 2017.

Among patients who received ≥1 antimicrobial, use of third-generation cephalosporins increased from 12.8% in 2002 to 17.5% in 2017 (*P* < .0001). Vancomycin use (oral and intravenous use combined) increased from 11.6% (95% CI, 10.3%−13.0%) in 2002 to 14.4% (95% CI, 13.2%−15.7%) in 2009 and remained stable at 12.6% (95% CI, 11.5%−13.8%) in 2017. The use of sulfonamides and trimethoprim combinations increased by 42.7% between 2002 and 2017 (from 6.6% to 9.4%, *P* = .0002). Carbapenem use increased by 57.6% between 2002 and 2009 (from 3.9% to 6.1%, *P* < .0001) and increased by 4.8% between 2009 and 2017 (*P* = .60). Daptomycin use increased from 0.03% in 2009 to 0.8% in 2017 (*P* = .001). Between 2002 and 2017, fluoroquinolone use decreased 36.5% (from 25.7% to 16.3%; *P* < .0001) and clindamycin use decreased 62.5% (from 25.7% to 16.3%; *P* < .0001). In addition, aminoglycoside use decreased from 8.8% to 2.4% (*P* < .0001) and metronidazole use decreased from 18.1% to 9.4% (*P* < .0001).

### Antimicrobial use by age category

**Fig. 3.**
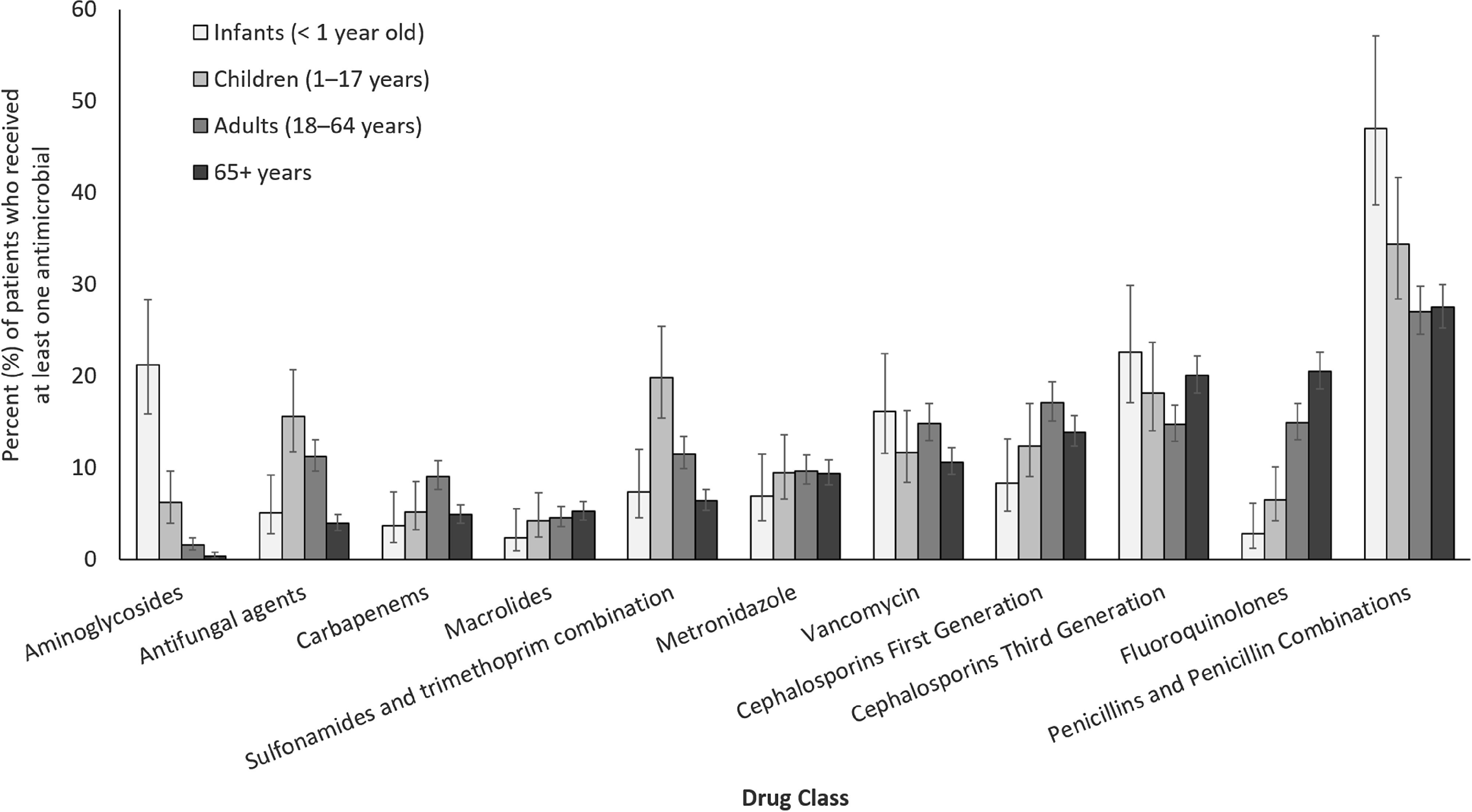
Percentage of patients in each age category who received selected antibiotic classes of all patients in the age category who received any antimicrobial agent in 2017 with 95% confidence intervals; antimicrobial classes ordered left to right from least to most frequently used overall.

The most frequently used antimicrobials varied by age category. The class of penicillins and penicillin combinations was the most commonly received class in each age category (Figure 3). Among infants (<1 year) who received ≥1 antimicrobial in 2017, the two most frequently used drug classes were penicillins and penicillin combinations (47.0%) and third-generation cephalosporins (21.2%). Among children (1–17 years) who received ≥1 antimicrobial, the two most frequently used classes were penicillins and penicillin combinations (34.4%) and sulfonamides and trimethoprim combinations (19.8%). Among adults 18–64 years, the two most frequently used classes were penicillins and penicillin combinations (27.0%) and first-generation cephalosporins (17.1%). In patients aged >65 years who received an antimicrobial, the two most frequently used classes were penicillins and penicillin combinations (27.5%) and fluoroquinolones (20.5%).

**Fig. 4.**
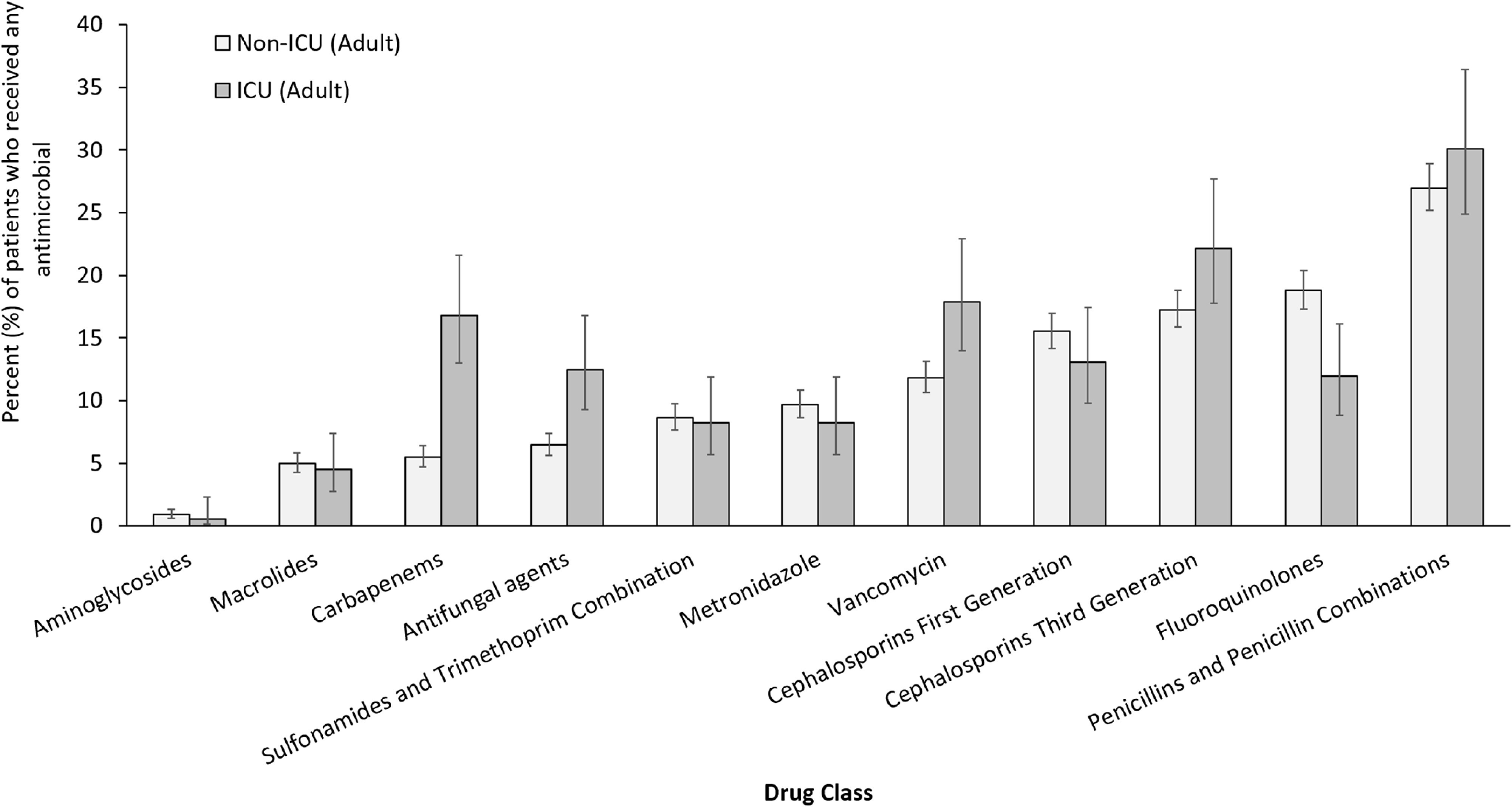
Percentage of adult patients in ICU or non-ICU who received selected antibiotic classes of all adult patients in ICUs or non-ICUs who received any antimicrobial agent in 2017 with 95% confidence intervals; antimicrobial classes ordered left to right from least to most frequently used overall.

### Secondary analysis

In the secondary analysis of the 18 hospitals that participated in all three surveys, the prevalence of antimicrobial use was similar to the primary analysis. Antimicrobial use increased from 36.3% (95% CI, 34.8%−37.8%) in 2002 to 39.2% (95% CI, 37.8%−40.6%) in 2009 and to 39.9% (95% CI, 38.5%−41.3%) in 2017.

## Discussion

We determined the prevalence of antimicrobial use among sentinel Canadian acute-care hospitals across three point-prevalence surveys performed in 2002, 2009, and 2017. Antimicrobial use significantly increased from 2002 and 2009, and stabilized between 2009 and 2017. This stabilization coincides with an increased focus on antimicrobial use and antimicrobial stewardship. Regional and national surveys of Canadian hospitals have found an increase in antimicrobial stewardship programs since the mid-2000s,^5–9^ with an increased focus on evaluating antimicrobial use quantitatively.^5,6^ In addition, in 2013, Accreditation Canada made antimicrobial stewardship a required organizational practice for acute-care hospitals.^10^

Across the surveys, 37%−40% of patients were receiving ≥1 antimicrobial during the 24-hour survey period. This range is similar to those reported in studies from other countries, although there are differences in methodologies and seasonal timing of the surveys. A 2015 point-prevalence survey conducted at 303 hospitals in 53 countries found that 34% (country range, 22%−86%) of adult inpatients had received ≥1 antimicrobial on the survey day.^11^ A 2011 one-day point-prevalence study conducted in 10 states in the United States reported that 50% of patients received ≥1 antimicrobial on either the day of survey or the day prior.^12^ A 2011−2012 European Centre for Disease Prevention and Control (ECDC) point-prevalence survey of 33 countries estimated that 33% of hospitalized patients receive ≥1 antimicrobial on any given day in the European Union and European Economic Area.^13^ In a subsequent ECDC survey conducted in 29 countries in 2016−2017, 33% of patients had received ≥1 antimicrobial on the survey day (country range, 16%−56%).^14^

We detected an increase in the percentage of adult ICU patients receiving just one antimicrobial from 26% of ICU patients in 2002 to 36% in 2017. Potential reasons for this include more patients receiving β-lactam/β-lactamase inhibitors or carbapenems instead of multiple narrower-spectrum antimicrobials, decreasing use of empiric vancomycin and metronidazole in combination with other antimicrobials, or (given the increases in more narrow spectrum agents such as trimethoprim-sulfamethoxazole) more frequent de-escalation to monotherapy following culture results. It is equally possible that multiple changes contributed.

Although the overall prevalence of antimicrobial use increased between 2002 and 2009 and stabilized between 2009 and 2017, different trends were identified among subclasses of drugs, emphasizing the importance of monitoring subclass use as well as absolute antimicrobial use. Based on available data, it was not possible to determine the reasons for the observed trends. Some potential drivers could include the development of antimicrobial stewardship programs, changes to antimicrobial prescribing guidelines, and changes in patient populations not captured through current survey methods. The observed decrease in metronidazole use could reflect greater use of oral vancomycin to treat CDI among hospitalized patients, a decrease in CDI incidence among hospitalized patients,^15^ or greater use of broad-spectrum antimicrobials that include anaerobic coverage (eg, β-lactam/β-lactamase inhibitor combinations or carbapenems).

Clindamycin and fluoroquinolone use have decreased. The risks of hospital-acquired CDI may have influenced physician willingness to prescribe these drugs because both are associated with CDI risk and, in the early 2000s in Quebec, major hospital-associated CDI outbreaks associated with fluoroquinolone use occurred.^16,17^ Additionally, since the late 2000s, there have been warnings of adverse events associated with fluoroquinolone use in the United States^18,19^ and Canada,^20–22^ which may have influenced the use of these antimicrobials. Notably, although the use of clindamycin and fluoroquinolones has decreased, there has been an overall increase in the prevalence of patients receiving antimicrobials with a high risk of CDI, largely due to increases in piperacillin-tazobactam use.

We detected a significant increase in the use of carbapenems, often reserved for high-risk settings or highly antibiotic resistant pathogens between 2002 and 2009. However, the prevalence of use stabilized between 2009 and 2017, potentially the result of successful antimicrobial stewardship.

Vancomycin use (oral and intravenous use combined) increased from 2002 to 2009 but stabilized between 2009 and 2017. We did not collect information on administration route, but previous work found that oral vancomycin represents ∼7% of vancomycin use in CNISP adult hospitals.^6^ Possible reasons for the stabilization in vancomycin use include the stabilization in MRSA infections at these hospitals,^3^ a decrease in the rate of CDI,^15,23^ and/or antibiotic stewardship programs discouraging and limiting empiric use of vancomycin for patients with lower risk of MRSA infection.^24^ The stabilization of vancomycin use could potentially be due to the availability of more recent alternative agents that are effective against antibiotic-resistant, gram-positive bacteria, such as daptomycin. Similar to previous studies, we observed a marked decline in aminoglycoside use, which is possibly due to the substitution of less nephrotoxic agents active against gram-negative bacteria.^25^

In this study, antimicrobial use within the CNISP hospital network was stable between the 2009 and 2017 surveys. This trend differed slightly from a previous CNISP study that identified a 12% reduction in antimicrobial use between 2009 and 2016, measured by defined daily doses (DDDs) per 1,000 person days per year in adult inpatients.^2^ The difference in trends may be due to study designs (eg, use prevalence vs DDDs, point prevalence among all inpatients vs annual dispensed antimicrobials among adult inpatients). Antimicrobial use estimates from point-prevalence surveys may have higher correlation with monthly DDD measurements than with annual DDDs.^26^ The latter two point-prevalence surveys involved 39 and 47 CNISP hospitals, respectively, whereas the analysis of antimicrobial use among adult inpatients represented 22 CNISP hospitals (18 hospitals participated in both studies). In both studies, we observed a significant decrease in the use of fluoroquinolone, metronidazole, and clindamycin and a significant increase in the use of third-generation cephalosporins.

### Lessons learned and limitations

Aside from the survey results themselves, the experience of implementing successive point-prevalence surveys across 15 years has generated important lessons for future surveys. The success of these surveys has depended on the active collaboration between the involved organizations via formal working groups and annual meetings as well as the informal sharing of experiences and expertise.

We did not collect data on indication of use, duration of use, dosage, route of administration, or whether multiple antibiotics were received concurrently or sequentially. Without these details, we were unable to assess appropriateness of use and it was more difficult to compare our results to results from surveillance programs that collect DDDs or days of therapy. Without information on indication for use, the reasons behind the observed trends are challenging to determine with confidence. For those considering implementing similar surveys, if resources allow, more detailed data could be collected from all or from a subset of participants. Additionally, it is important to consider generalizability in sentinel surveillance systems; hospitals having the resources and motivation to participate in surveillance initiatives likely differ from other hospitals. The hospitals included in these surveys were primarily large, urban, tertiary, acute-care facilities; thus, our findings may not be generalizable to all Canadian inpatient populations. Since 2017, our network has made additional efforts to recruit and support the participation of smaller community hospitals and hospitals from more rural regions.

Our surveys have a few additional limitations. By focussing on class of antibiotics, we may miss trends within specific antibiotics. The list of approved antimicrobials changes over time which can make interpretation of trends more difficult. Also, we were not able to assess all possible changes in patient mix between the surveys. Furthermore, we may not have captured sufficient data to identify underlying changes in the patient population in Canadian hospitals. It has been reported anecdotally that hospitalized patients at these hospitals in 2017 were, on average, more acutely ill than in 2002. If patients in 2017 had an increased need for antibiotics due to more complex procedures and invasive devices putting them at higher risk of infection, the recent stabilization of antimicrobial use is a promising trend.

In conclusion, we detected an increase in the prevalence of antimicrobial use between 2002 and 2009, followed by stabilization between 2009 and 2017. We observed changes in use for individual antimicrobial classes or agents, notably declines in clindamycin and fluoroquinolone use. Further studies are needed to examine the appropriateness of antimicrobial use, as part of a coordinated approach to prevent the emergence and spread of antimicrobial resistance.

## Supplementary material

For supplementary material accompanying this paper visit https://doi.org/10.1017/ice.2021.519. click here to view supplementary material
