# Supplementary material for: Antimicrobial use in Canadian acute-care hospitals: Findings from three national point-prevalence surveys between 2002 and 2017
Source: Infect Control Hosp Epidemiol. 2022 Mar 7;43(11):1558–64. doi: 10.1017/ice.2021.519 (PMC9672830; doi:10.1017/ice.2021.519)
Supplement: Supplementary file 1 [file S0899823X21005195sup.zip › S0899823X21005195sup001.docx]

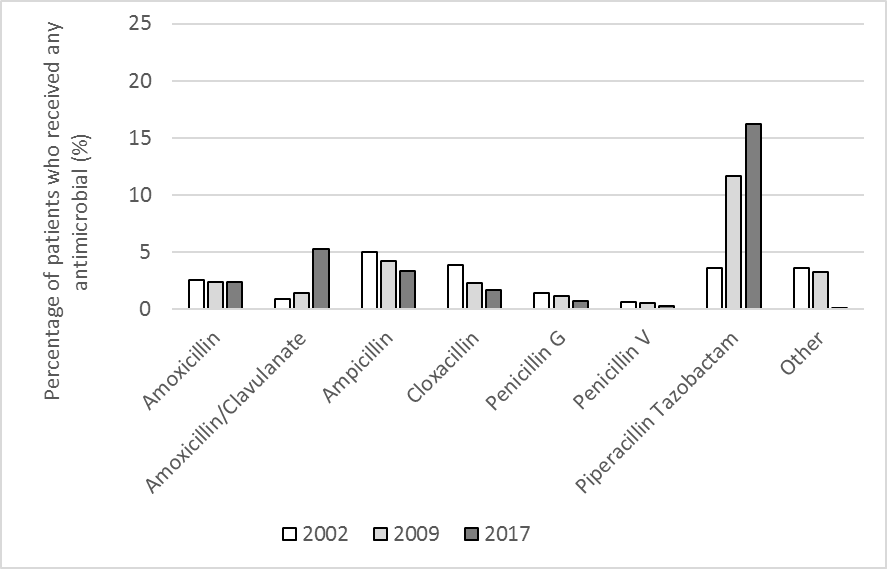


Supplemental Figure 1. Percentage of patients who received specific penicillin or penicillin combination class antibiotics out of all patients who received any antimicrobial agent in 2002, 2009, 2017
