# Supplementary material for: Antimicrobial use in Canadian acute-care hospitals: Findings from three national point-prevalence surveys between 2002 and 2017
Source: Infect Control Hosp Epidemiol. 2022 Mar 7;43(11):1558–64. doi: 10.1017/ice.2021.519 (PMC9672830; doi:10.1017/ice.2021.519)
Supplement: Supplementary file 1 [file S0899823X21005195sup.zip › S0899823X21005195sup002.docx]

Supplemental Table 1: Antimicrobial agents collected in the three point prevalence surveys

| **Classification** | **Generic Name** |
| --- | --- |
| Penicillins and combinations of penicillins | Amoxicillin |
|  | Amoxicillin/Clavulanate |
|  | Ampicillin |
|  | Cloxacillin |
|  | Nafcillin |
|  | Penicillin G |
|  | Penicillin V |
|  | Piperacillin |
|  | Piperacillin Tazobactam |
|  | Ticarcillin/Clavulanate |
| Carbapenems | Imipenem |
|  | Meropenem |
|  | Ertapenem |
| Aminoglycosides | Amikacin |
|  | Gentamicin |
|  | Tobramycin |
| Cephalosporins First Generation | Cefadroxil |
|  | Cefazolin |
|  | Cefalexin |
|  | Cefalotin |
| Cephalosporins Second Generation | Cefaclor |
|  | Cefonicid |
|  | Cefoxitin |
|  | Cefuroxime |
| Cephalosporins Third Generation | Cefixime |
|  | Cefotaxime |
|  | Ceftazidime |
|  | Ceftizoxime |
|  | Ceftobiprole |
|  | Ceftriaxone |
| Cephalosporins Fourth Generation | Cefepime |
| Cephalosporin/beta-lactamase inhibitor combinations | Ceftazidime/Avibactam |
|  | Ceftolazane/Tazobactam |
| Macrolides | Azithromycin |
|  | Clarithromycin |
|  | Erythromycin |
| Fluoroquinolones | Ciprofloxacin |
|  | Norfloxacin |
|  | Levofloxacin |
|  | Moxifloxacin |
|  | Gatifloxacin |
| Antifungal agents | Amphotericin B |
|  | Fluconazole |
|  | Posaconazole |
|  | Voriconazole |
|  | Itraconazole |
|  | Caspofungin |
|  | Micafungin |
|  | Anidulafungin |
|  | Other antifungal |
| Antituberculous Medications | Ethambutol |
|  | Isoniazid |
|  | Pyrazinamide |
|  | Rifampicin |
|  | Other antituberculous medications |
| Tetracyclines | Tetracycline |
|  | Doxycycline |
|  | Tigecycline |
| Sulfonamides and trimethoprim combinations | Sulfamethoxazole/Trimethoprim |
|  | Sulfadiazine/Trimethoprim |
| Others | Clindamycin |
|  | Metronidazole |
|  | Nitrofuratoin |
|  | Vancomycin |
|  | Daptomycin |
|  | Linezolid |
|  | Colistin |
|  | Aztreonam |
|  | Others |
| Antivirals | Oseltamivir |
|  | Other antiviral medications |
